# Study of High Performance Sulfonated Polyether Ether Ketone Composite Electrolyte Membranes

**DOI:** 10.3390/polym11071177

**Published:** 2019-07-12

**Authors:** Gwomei Wu, Sheng-Jen Lin, I-Chan Hsu, Juin-Yih Su, Dave W. Chen

**Affiliations:** 1 Institute of Electro-Optical Engineering, Department of Chemical and Materials Engineering, Chang Gung University, Taoyuan 333, Taiwan; 2 Chang Gung Memorial Hospital, Keelung 204, Taiwan

**Keywords:** composite electrolyte membrane, polyether ether ketone, sulfonation, nano SiO_2_, supercritical carbon dioxide

## Abstract

In this study, high performance composite electrolyte membranes were prepared from polyether ether ketone polymeric material. An initial sulfonation reaction improved the membrane hydrophilicity and its water absorbability and thus enhanced the ionic conductivity in electrochemical cells. Protonic conductivity was improved from 10^−4^ to 10^−2^ S cm^−1^ with an increasing sulfonation time from 72 to 175 h. The effects of blending nano SiO_2_ into the composite membranes were devoted to improve thermal and mechanical properties, as well as methanol permeability. Methanol permeability was reduced to 3.1 × 10^−7^ cm^2^ s^−1^. Finally, a further improvement in ionic conductivity was carried out by a supercritical carbon dioxide treatment under 20 MPa at 40°C for 30 min with an optimum SiO_2_ blend ratio of 10 wt-%. The plasticizing effect by the Lewis acid-base interaction between CO_2_ and electron donor species on polymer chains decreased the glass transition and melting temperatures. The results show that sulfonated composite membranes blended with SiO_2_ and using a supercritical carbon dioxide treatment exhibit a lower glass transition temperature, higher ionic conductivity, lower methanol permeability, good thermal stability, and strong mechanical properties. Ionic conductivity was improved to 1.55 × 10^−2^ S cm^−1^. The ion exchange capacity and the degree of sulfonation were also investigated.

## 1. Introduction

Due to rising costs in natural energy resources and growing concerns for environmental protection, modern society has been in great demand for more efficient energy storage and consumption technologies [1,2,3,4]. However, the production of usable energy from the combustion of fuels is an inefficient process due to intrinsic thermodynamic principles. A modern electric power plant is unable to harness more than 35–40% of the potential energy from oil, coal, or natural gases. A gasoline engine has an efficiency of only about 25–30%. The rest of the energy is inevitably lost to the surroundings as heat waste. An additional cooling system is thus required to solve this problem. Fuel cells are electrochemical cells that consume fuels under nearly thermodynamically reversible conditions [5,6]. The energy conversion efficiencies are thus much higher. Fuels can be fed continuously to produce power. This operation is not only more efficient but also more economically feasible. Therefore, many researchers are making large amounts of effort to find the best materials and processes to elevate the electrochemical performance of fuel cells for different applications [7,8,9,10].

In order to obtain higher energy and power density for fuel cells, a solid polymer electrolyte membrane (SPEM) can be used to replace the heavy-weight aqueous electrolytes, such as non-woven polypropylene/polyethylene, Nafion^®^, and Flemion^®^. Nafion^®^ has been used as a separator in some fuel cells, but its cost is quite high [11]. For the practical application of an SPEM in direct methanol fuel cells, it is necessary to have low methanol permeability, high ionic conductivity, good mechanical strength, good thermal stability, and lower cost [12,13]. In the last decade, polyether ether ketone (PEEK) has exhibited great potential to be developed as an SPEM due to its balanced combination properties of good mechanical strength, high thermal stability, and superior chemical resistance. The hydrophobic property and the high glass transition temperature (T_g_) of PEEK are intrinsic and need to be improved for SPEM preparation. For direct methanol fuel cell and proton exchange membrane fuel cell applications, it is beneficial to be more hydrophilic. As such, more water electrolytes can be sustained in the free volume of the matrix. This will allow for easier ionic transport during electrochemical cell discharge. Some studies have focused on blendings with second polymers, such as polyether sulfone, polyphenyl sulfone, polyphenylene sulfide, and polyvinyl pyrrolidone [14,15]. Further improvements are still desired for their applicability. On the other hand, it has been reported that polyaryl ether ketones under sulfonation—instead of perfluorinated polymers—were quite durable under fuel cell conditions [16,17]. PEEK has many unique characteristics that could have great potential for future use in polymer electrolyte fuel cells [18,19].

This study reveals an improved method to increase the hydrophilicity of PEEK by suitable sulfonation in concentrated sulfuric acid at a long time and a high temperature. The sulfonated PEEK (sPEEK) polymer can be incorporated with nano particles to improve mechanical strength and ionic conductivity [20,21]. A supercritical CO_2_ (scCO_2_) fluid treatment can be further adapted to reduce its T_g_. The CO_2_ molecules have a plasticizing effect by the Lewis acid-base interaction between the CO_2_ and the electron donor species on the polymer chains [22,23]. Both the T_g_ and melting temperature (T_m_) of the polymer can be reduced. scCO_2_ treatment in polyethyleneoxide-LiCF_3_SO_3_ has been studied for use in lithium-salt polymer electrolytes and has shown significant improvement in ionic conductivity [24,25].

In this paper, we prepared high performance sulfonated PEEK composite electrolyte membranes with high ionic conductivity, low methanol permeability, good mechanical strength, and good stability. The high hydrophilicity and water absorbability of sPEEK was introduced by sulfonation that reacted in concentrated sulfuric acid solution. The ion exchange capacity (IEC) and the degree of sulfonation (DS) were also investigated. The sPEEK membranes were modified by blending with nano scale SiO_2_ particles at different weight ratios from 1 to 20 wt-%. The sPEEK and sPEEK/SiO_2_ composite membranes were further treated in supercritical carbon dioxide under the condition of 20 MPa at 40 °C. In addition, the methanol permeability for the composite electrolyte membranes was also carefully investigated.

## 2. Materials and Methods

**Figure 1:**
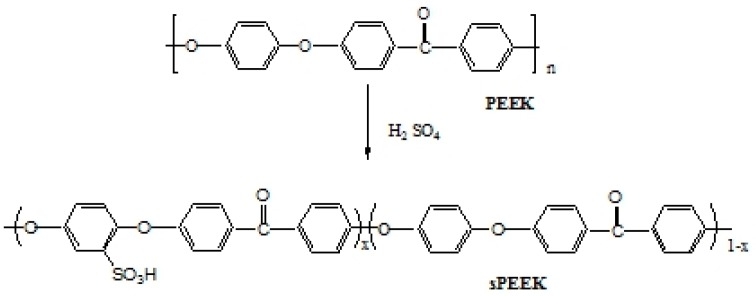
Chemical structures of polyether ether ketone (PEEK) and sulfonated PEEK(sPEEK)in the sulfonation reaction.

Polyether ether ketone (PEEK, Victrex US, Inc., Grade 450G, M.W. 34,000, West Conshohocken, PA, USA) was dried and used for a sulfonation reaction. The sulfonation reaction was carried out by dissolving the PEEK granules in 98% concentrated sulfuric acid (Aldrich, St. Louis, MO, USA). The polymer/H_2_SO_4_ ratio was 0.05 g/mL. The reaction was conducted in continuous mechanical stirring at 90°C from 72 h to 175 h. After the reaction, the maroon color solution was quickly quenched in iced, de-ionized water. The white-color precipitate of sulfonated PEEK then appeared. The sPEEK precipitate was rinsed in fresh de-ionized water for several times until the water became neutral. The sulfonated sample was dried in a vacuum oven at 60 °C for more than 1 day. Figure 1 shows the chemical structures of PEEK and sPEEK, while the x denotes the extent of the sulfonation reaction.

The dried sPEEK polymer was firstly dissolved in a *N*-methyl-2-pyrrolidone (NMP, Aldrich) solvent. The appropriate weight of nano SiO_2_ (Aldrich, 10 nm, 640 m^2^/g) was also added to the NMP solvent in another beaker under ultrasonic vibration for at least 90 min. The completely dissolved solution of sPEEK/NMP was then mixed with SiO_2_/NMP under continuous mechanical agitation for 36 h. The mixture solution was ultrasonically vibrated for 120 min before its casting on a glass plate. The sample was then dried in a vacuum oven at 90°C for 2 days to remove the residual solvent. The completely dried sPEEK/SiO_2_ composite membrane was further treated in supercritical CO_2_ fluid using the Apeks Supercritical extraction system with a flow rate of 2 mL min^−1^ in the reactor at 40 °C and 20 MPa for 30 min. All the samples were then slowly cooled to the ambient temperature to obtain the transparent composite membranes.

The glass transition temperatures of the membranes were analyzed by differential scanning calorimetry (DSC, TA 2010, New Castle, DE, USA) purged by N_2_ gas, and the temperature was increased to 250°C at a heating rate of 10°C/min. The AC impedance method was employed to measure ionic conductivity. The sPEEK polymeric composite electrolyte membranes were sandwiched between SS316 stainless steel electrodes with the surface area of 0.785 cm^2^. The AC impedance measurements were carried out by AutoLab from Eco Chemi with a computer program. The frequency range was from 100 Hz to 100 kHz, and an excitation signal of 10 mV was used. In addition, a convection oven was set up to control the testing temperature. The following equation was used to calculate the ionic conductivity in this study.

(1) $$\sigma =\frac{L}{R_{b}\times A}$$

where *L* and *A* are the thickness and area of the composite membranes, respectively. *R_b_* was derived from the Nyquist plot of the AC impedance analysis at a low intersection of the high frequency semi-circle on a complex impedance plane on the z axis.

The methanol permeability of the sPEEK polymer composite membranes was measured using a glass diffusion cell. The cell consisted of two compartments with a circular hole of 3 cm in diameter. One compartment was filled with a 35 mL solution of 3 M methanol, and the other compartment was filled with 35 mL of de-ionized water. The solution in each compartment was continuously stirred to keep uniform concentration, and the methanol concentration of each cell was measured by gas chromatography (GC, Varian, Palo Alto, CA, USA). The measurement was performed in a water bath at 30 °C, and the area of the membrane was controlled at 5.3 cm^2^.

For the analysis of water absorption, the fully dried composite membrane samples with an area of 3 × 3 cm^2^ were first weighed (W_0_) and then immersed in de-ionized water for 2 days. The soaked membrane surface was wiped with filter paper, and the wet membrane weight was obtained (W_1_). The water absorption (A%) was calculated from the following equation:

(2) $$A\%=\frac{W_{1}-W_{0}}{W_{0}}$$

The ion-exchange capacity (IEC) of the s-PEEK composite electrolyte membranes was analyzed by a titration method. It was determined by equilibrating the membranes in a 1.0 M HCl solution for 24 h so that the charge group of the membrane was in H^+^ form. The membrane was then washed with de-ionized water free of HCl before equilibration in 0.1 M NaCl for 24 h. Finally, the ion-exchange capacity was determined by the back titration method with a 0.1 M NaOH solution. The degree of sulfonation (DS) was also determined from the IEC data:

(3) $$x=\frac{M_{w,p}IEC}{1-M_{w,f}IEC}$$

where *M*_*w*,*p*_ is the molecular weight of the nonfunctional polymer repeat unit and *M*_*w*,*f*_ is the molecular weight of the functional group with the counter ion (–SO_3_Na).

## 3. Results and Discussion

### 3.1. IEC and DS Analysis

**Figure 2:**
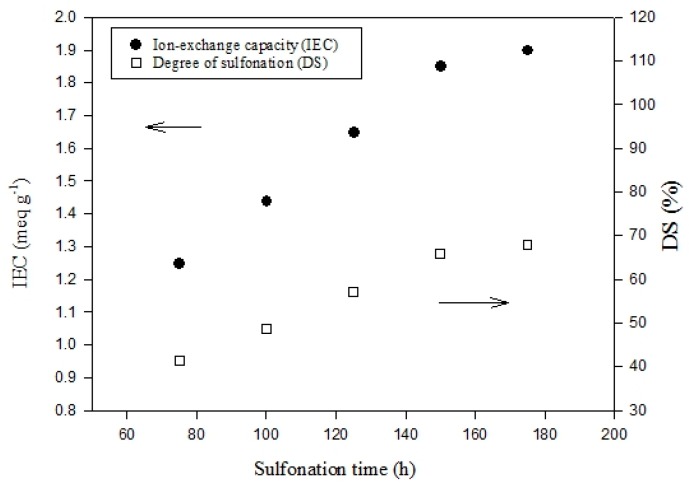
Ion exchange capacity (IEC) and degree of sulfonation (DS) analysis results of the sPEEK membranes with different sulfonation reaction times at 90°C. At 175 h, the IEC was 1.9 meq g^−1^ and DS was 68%.

**Figure 3:**
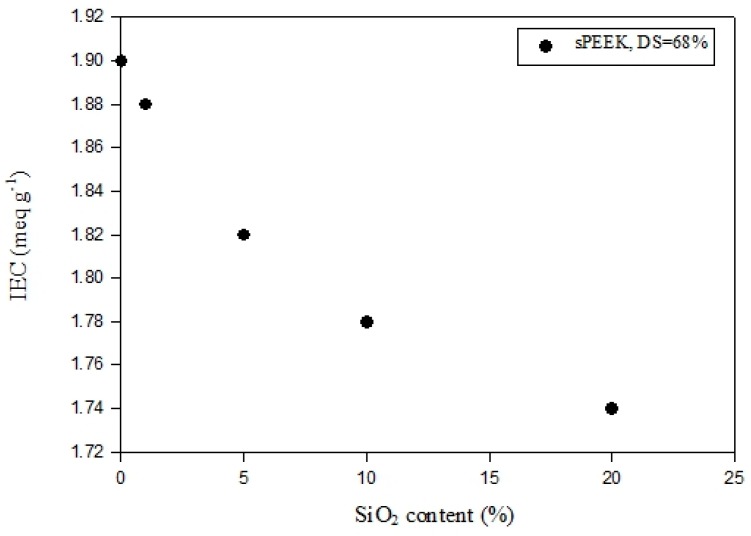
Effects of nano SiO_2_ content on composite membrane IEC. The DS was 68% for the sPEEK polymer.

Figure 2 shows the IEC and DS analysis results of the sPEEK membranes with different sulfonation reaction times in the range of 72–175 h. It is clearly indicated that both the IEC and DS values continuously increased with the increasing sulfonation time. However, the increase rate appeared to slow down when over 150 h, indicating the approaching saturation of the reaction. Nevertheless, the IEC and DS reached up to 1.9 meq g^−1^ and 68%, respectively. The effects of nano SiO_2_ content for sPEEK composite membranes with the DS fixed at 68% are provided in Figure 3. While the SiO_2_ content increased from 0 to 20 wt-%, the IEC value decreased from 1.90 to 1.74 meq g^−1^. This can be attributed to the interaction of hydrogen bonding between the nano SiO_2_ particles and the sulfonic groups of sPEEK. The ion exchange capability was thus reduced accordingly.

### 3.2. IR Analysis

**Figure 4:**
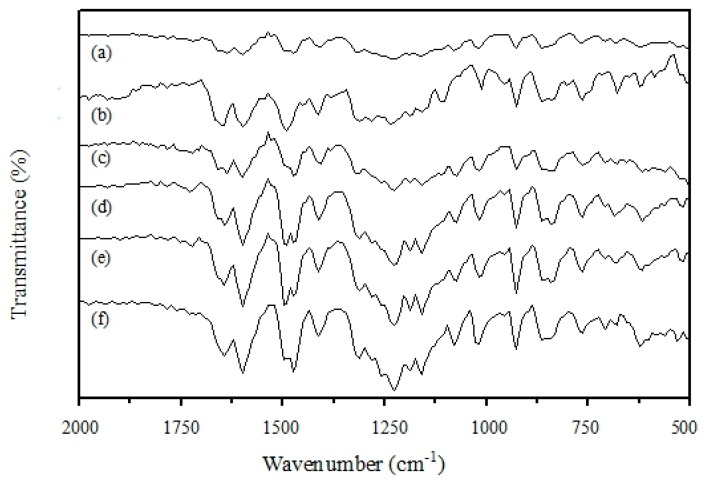
FTIR spectra for the sulfonated PEEK samples with different degrees of sulfonation: (**a**) 0%, (**b**) 41%, (**c**) 49%, (**d**) 57%, (**e**) 66%, and (**f**) 68%.

The Fourier-transform infrared (FTIR, Jasco) spectra for the sulfonated PEEK samples with different DS values from 0% to 68% are shown in Figure 4. There are three characteristic absorption bands of –SO_3_H groups that appear in the wave number region of 1020 cm^−1^, 1250 cm^−1^, and 1650 cm^−1^. These absorption bands can be assigned to the S=O stretch, the asymmetric O=S=O stretch, and the symmetric stretching vibration of O=S=O due to the –SO_3_H groups, respectively [26]. The substitution chemical reaction took place in one of the four positions of the aromatic rings between the ether groups. This was caused by the relatively lower electron density of the other two aromatic rings in the polymer’s monomer structure. The electron-attracting nature from the neighboring carboxyl group played a role [16,18]. The IR analysis results, in conjunction with the previous IEC analysis, have confirmed that the sulfonic functional groups were indeed quantitatively introduced to the PEEK aromatic polymer molecules.

### 3.3. Water Absorption and Ionic Conductivity

Table 1 summarizes the effects of sulfonation time on water absorption, ionic conductivity, and the degree of sulfonation for the sPEEK membrane samples. The water absorption increased with the increasing sulfonation time. This was due to the improvement in hydrophilicity by chemically incorporating the sulfonic groups into the polymer main chains. Therefore, the water content in sPEEK membranes became higher. This induced water channel in the free volume of the polymer matrix among the sulfonic groups, which then provided a smooth pathway for ionic transport. The ionic conductivity was thus significantly improved to 4.68 × 10^−3^ S cm^−1^ for the 68% degree of sulfonation (DS68) sPEEK membrane sample, and the water absorption was 45.3%. On the other hand, both the effects of the nano SiO_2_ blend ratio and the scCO_2_ treatment results on the polymer composite electrolyte membranes are shown in Table 2. The sPEEK blends with low SiO_2_ contents were unable to elevate the water absorbability and the ionic conductivity. This was shown by the high hydrogen bonding between the SiO_2_ and –SO_3_H groups, and the SiO_2_ particles occupied a finite free volume which reduced the space for water content. However, while the nano SiO_2_ content increased to more than 10 or 20 wt-%, more SiO_2_ nano-particles could loosen the tight polymer chains and sustain more water content. Thus, ionic conductivity and water absorbability could be elevated. When the sPEEK/SiO_2_ composite membranes were conducted in a supercritical CO_2_ fluid treatment, the interaction between SiO_2_ and the sulfonic groups was destroyed, and the SiO_2_ particles apparently loosened the tight polymer chains and largely increased the free volume to contain more water. Therefore, the water absorption and ionic conductivity for the composite membranes (sample SCF-DS68-S10) were both improved significantly to 63.1% and 1.55 × 10^−2^ S cm^−1^, respectively.

### 3.4. DSC and TGA Thermal Analysis

**Figure 5:**
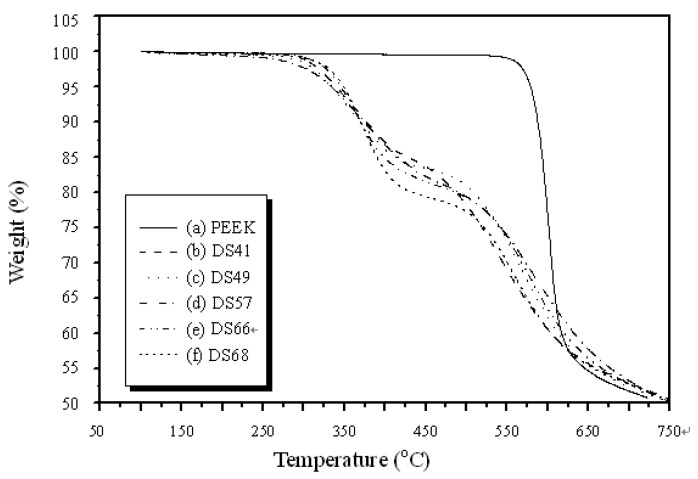
Thermal gravimetric analysis (TGA) thermographs of the original PEEK and sPEEK membrane samples.

**Figure 6:**
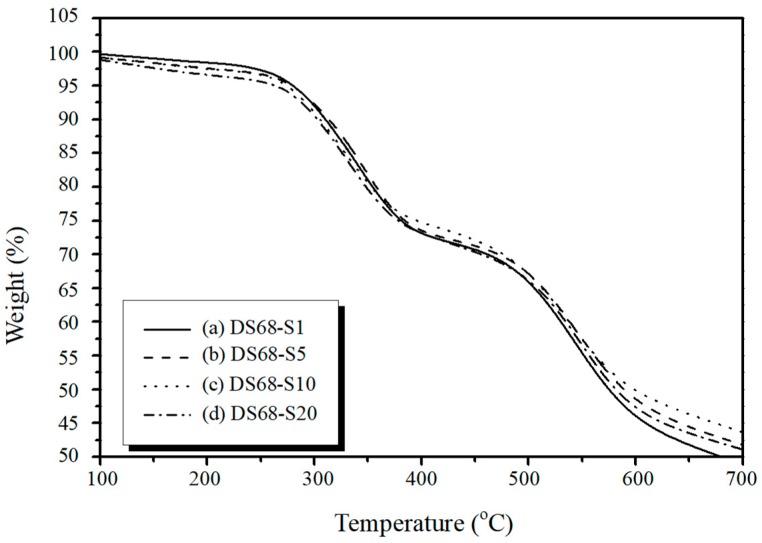
TGA thermographs of the sPEEK/SiO_2_ composite membranes with a 68% degree of sulfonation (DS68). The nano SiO_2_ content varied at 1% (S1), 5% (S5), 10% (S10), and 20% (S20).

The thermal gravimetric analysis (TGA) thermographs of the sPEEK membrane samples are shown in Figure 5. The original PEEK polymer exhibited one-step degradation without the sulfonation. The derivative scan had a peak loss at around 600°C. For the highly sulfonated sPEEK membranes, a two-step thermal degradation process was observed. There was a weight loss of about 10–20% at a temperature range of 300–380°C when the degree of sulfonation was increased to 68%. However, this was close to the melting temperature of PEEK (T_m_ = 350 °C). In addition, Figure 6 shows the TGA thermographs of the sPEEK/SiO_2_ composite membranes while the DS was fixed at 68%. For the higher nano SiO_2_ content in sPEEK from 1% to 20%, the weight loss at this temperature range increased to about 30%. The later decomposition temperature was observed from the peak loss of the derivative scan at 500–600°C. Therefore, the sulfonation and nano SiO_2_ in sPEEK was able to slightly reduce the thermal degradation resistance for the sPEEK membrane after prolonged treatment.

**Figure 7:**
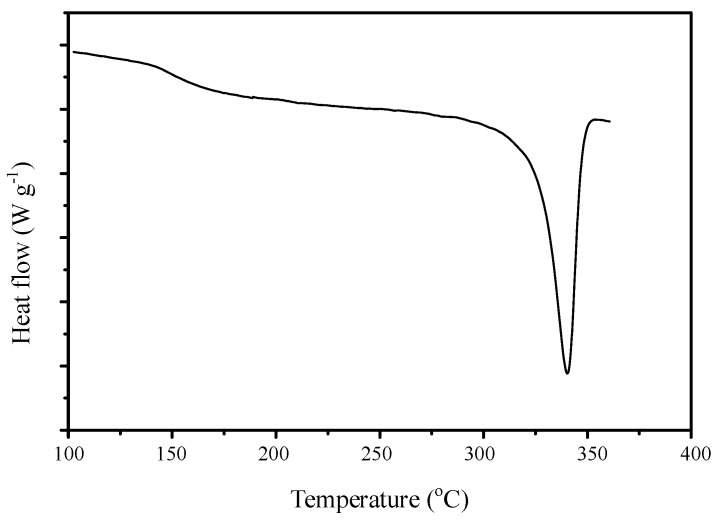
Typical differential scanning calorimetry (DSC) curve for the original PEEK. T_m_ was 340°C, and the T_g_ onset started at about 145°C.

**Figure 8:**
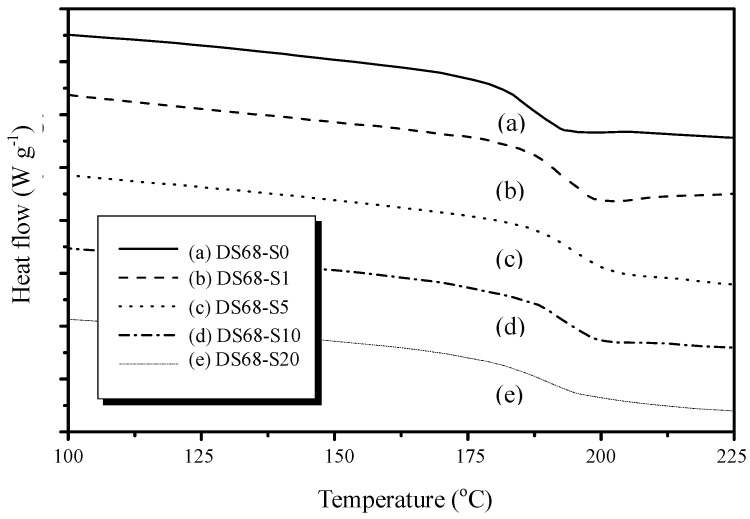
DSC curves for the sPEEK composite membranes with different SiO_2_ contents. The sulfonated sPEEK exhibited much higher T_g_ than the original PEEK polymer. The incorporation of nano particles increased T_g_ even more.

**Figure 9:**
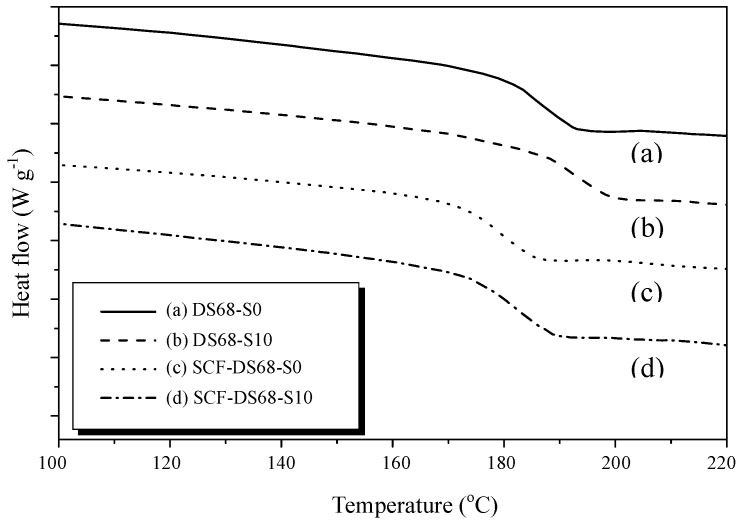
Effects of supercritical CO_2_ fluid (SCF) treatment on sPEEK/SiO_2_ composite membranes. T_g_ has been decreased on the DSC curves.

The typical DSC curve for the PEEK membrane is displayed in Figure 7. PEEK is a polymer with semi-crystalline structure, and the T_m_ can be seen at 340°C. The T_g_ onset starts at about 145°C. In order to verify the T_g_ for sPEEK composite samples with the different nano SiO_2_ contents, we narrowed down the temperature scan range to 100–225°C. From Figure 8, it can be clearly observed that the T_g_ for sPEEK with a 68% DS (sample DS68-S0) was about 190°C. It was much higher than that of the original PEEK polymer. With the incorporation of the nano SiO_2_ particles in the composite samples (DS68-S1~DS68-S20), the T_g_ was slightly further increased to 195°C due to the interaction of hydrogen bonding between SiO_2_ hydration cluster and the sulfonic groups. Figure 9 shows the effects of the super critical CO_2_ fluid (SCF) treatment using sPEEK with a DS of 68% and the SiO_2_ content of 10 wt-%. It is clearly evidenced that the T_g_ was significantly decreased from 195°C (sample DS68-S10) to183°C (sample SCF-DS68-S10). The interaction between SiO_2_ and the sulfonic groups was efficiently destroyed, and the SiO_2_ particles could just loosen the tight polymer chains, thus lowering the T_g_.

### 3.5. Mechanical Properties

**Figure 10:**
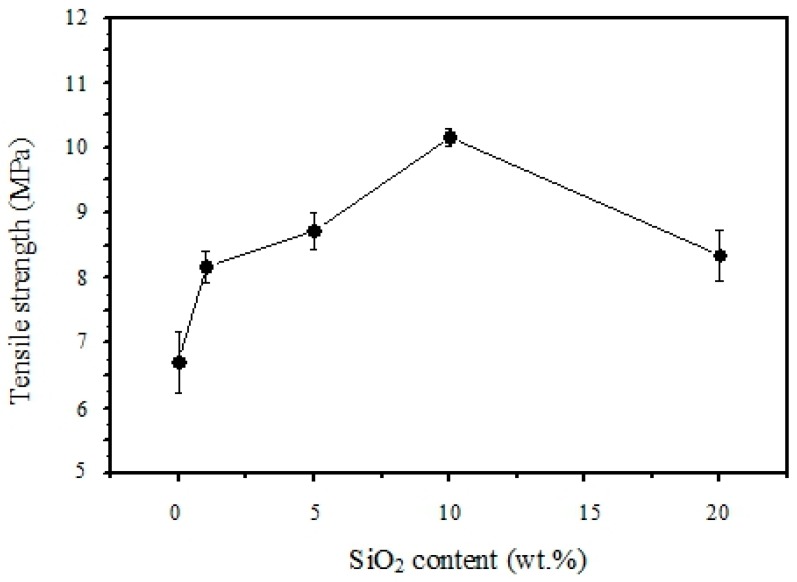
Effects of SiO_2_ content on the mechanical tensile strength of composite membranes at room temperature.

**Figure 11:**
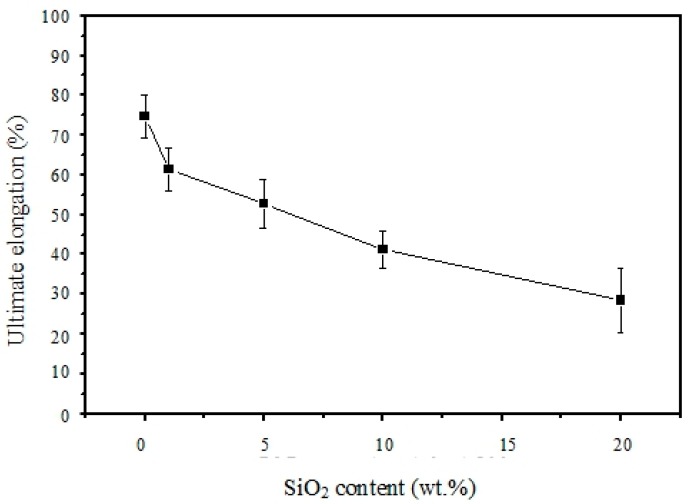
Elongation at-break results of the composite membrane samples at room temperature.

The mechanical strength and elongation properties were determined by a tensile stress-strain testing system (Instron) at room temperature, and the results are shown in Figure 10 and Figure 11. In general, the tensile strength of the polymer composite membrane increased with increasing SiO_2_ content, while the ultimate elongation at break decreased with it. The highest tensile strength of 10.3 MPa was found with 10 wt-% SiO_2_ content in the polymer composite membrane. The pure sPEEK membrane, without SiO_2_ content, exhibited a lower tensile strength of 6.5 MPa but with a good elongation-at-break value of 76%. However, the tensile strength was reduced to 8.3 MPa when the SiO_2_ content was as high as 20 wt-%. Its elongation-at-break was also further down to 28%. This could be attributed to the interaction of the hydrogen bonding between SiO_2_ and the sulfonic groups, and the tight polymer chains were partially destroyed. The interface played some role in the mechanical properties. In addition, it was noted that, after the sulfonation, the mechanical strength decreased due to the hydrophilicity of the sulfonic groups. The absorbed water plasticized the polymeric membranes [27].

### 3.6. Methanol Permeability

**Figure 12:**
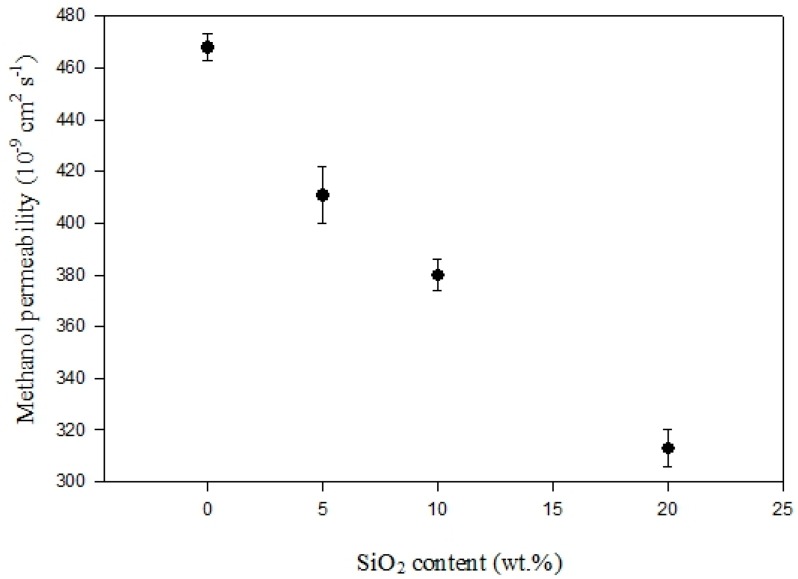
Methanol permeability data of the sPEEK/SiO_2_ composite electrolyte membranes with different SiO_2_ contents at 25 °C.

Figure 12 shows the methanol permeability analysis results through the sPEEK composite electrolyte membranes as a function of SiO_2_ content at 25°C. The DS was at 68%. The methanol permeability clearly decreased with the increasing SiO_2_ content. It was 0.47 × 10^−6^ cm^2^ s^−1^ for the sPEEK membrane (DS68-S0, without SiO_2_) and 0.31 × 10^−6^ cm^2^ s^−1^ for the composite membrane (DS68-S20, SiO_2_ content 20 wt-%). The methanol permeability data were all much lower than that of the Nafion^®^ 117 membrane, which were calculated at about 2 × 10^−6^ cm^2^ s^−1^ [28]. The sPEEK exhibited a higher mechanical strength, and the rigid benzene rings in the polymer chains provided a complex route for methanol permeation. With the SiO_2_ incorporation in the sPEEK membrane, the more complicated route reduced methanol permeation and was indeed very beneficial for the direct methanol fuel cell and proton exchange membrane fuel cell applications. The lower methanol permeability of the sPEEK composite electrolyte membranes could prolong the cycle life of fuel cells.

### 3.7. EDS Analysis

**Figure 13:**
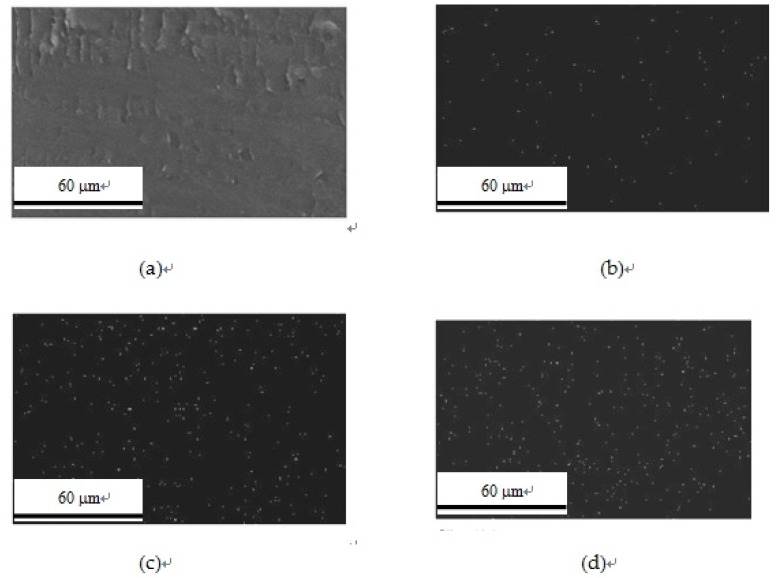
Energy dispersive X-ray spectroscopy(EDS) mapping micrographs based on the Si atom for the composite membranes with different nano SiO_2_ contents: (**a**) 0%, (**b**) 1%, (**c**) 10%, (**d**) 20%.

The dispersion of nano SiO_2_ particles in the sPEEK membranes was observed by using energy dispersive X-ray spectroscopy (EDS). The peak intensity of the Si atom increased with increasing nano SiO_2_ content from 1 wt-% to 20 wt-%. From the EDS mapping (Figure 13), it can be suggested that the nano SiO_2_ particles were well dispersed in the sPEEK membranes. Though a slight aggregation phenomenon of nano particles occurred as the SiO_2_ content increased to 20 wt-%, the uniform dispersion of SiO_2_ was still observed when the content was lower than 10 wt-%. The ultrasonic vibration for the mixing of SiO_2_ in sPEEK during the preparation step was necessary to warrant the well dispersion results. The uniformity of electrolyte membrane properties could thus be maintained for fuel cell applications.

## 4. Conclusions

We presented a simple method to prepare a novel proton solid polymer electrolyte with a high ionic conducting membrane and low methanol permeability. This was conducted by using a polyether ether ketone material via sulfonation and then blended with nano SiO_2_, along with a further supercritical CO_2_ fluid treatment to improve its characteristics. An infrared study verified the proper incorporation of sulfonic acid groups. The degree of sulfonation was further evidenced by the ion-exchange capacity determined by titration. The characteristic properties of the composite electrolyte membranes were carefully studied using DSC, TGA, EDS, an Instron tester, and AC impedance spectroscopy. The sulfonation process improved water absorption and ionic conductivity. The SiO_2_ incorporation also influenced ionic conductivity, and it was improved to 0.0073 S cm^−1^. This improvement was attributed to both loosening in rigid polymer chains and the more amorphous characteristic of the membranes. With a higher SiO_2_ content, a more complicated route was able to reduce methanol permeation and become very beneficial for direct methanol fuel cell and proton exchange membrane fuel cell applications. The composite sample with 68% DS and 10 wt-% SiO_2_ showed a low methanol permeability of 0.38 × 10^−6^ cm^2^ s^−1^ but a high mechanical strength of 10.3 MPa. The super critical CO_2_ fluid treatment was a useful process to improve the electrochemical characteristics for the composite electrolyte membranes. It was able to partially destroy the interaction between SiO_2_ and the sulfonic groups, which improved the ionic conductivity and water absorbability significantly to 1.55 × 10^−2^ S cm^−1^ and 63.1%, respectively. Consequently, it became possible to achieve a good balance between ionic conductivity and other important application properties. Further developments are still desired to meet requirements for different electrochemical devices.

## Figures and Tables

**Table 1:** Effects of sulfonation time on water absorption, ionic conductivity, and degree of sulfonation for the sPEEK membrane samples.

Sample	Membrane Properties			
	Sulfonation Time (h)	DS (%)	Absorption (%)	Ionic Conductivity (× 10^−3^ S cm^−1^)
DS41	72	41.0	18.2	0.05
DS49	96	48.7	24.9	0.26
DS57	120	57.2	32.0	1.29
DS66	144	65.8	36.1	2.62
DS68	175	68.0	45.3	4.68

**Table 2:** Effects of nano SiO_2_ blend ratio and supercritical CO_2_ fluid treatment on the 68% degree of sulfonation (DS68) sPEEK composite electrolyte membranes.

Sample	Properties				
	Sulfonation Time (h)	SiO_2_ Content (%)	Absorption (%)	Ionic Conductivity (× 10^−3^ S cm^−1^)	Supercritical CO_2_ Fluid Treatment
DS68-S0	175	0	45.3	4.68	No
DS68-S1	175	1	44.7	3.37	No
DS68-S5	175	5	42.0	3.18	No
DS68-S10	175	10	52.6	6.46	No
DS68-S20	175	20	59.2	7.34	No
SCF-DS68-S0	175	0	56.5	9.14	Yes
SCF-DS68-S10	175	10	63.1	15.47	Yes
